# MRI quantification of pancreas motion as a function of patient setup for particle therapy —a preliminary study

**DOI:** 10.1120/jacmp.v17i5.6236

**Published:** 2016-09-08

**Authors:** Giulia Fontana, Marco Riboldi, Chiara Gianoli, Cezarina I. Chirvase, Gaetano Villa, Chiara Paganelli, Paul E. Summers, Barbara Tagaste, Andrea Pella, Piero Fossati, Mario Ciocca, Guido Baroni, Francesca Valvo, Roberto Orecchia

**Affiliations:** ^1^ Clinical Bioengineering Unit, Centro Nazionale di Adroterapia Oncologica Pavia Italy; ^2^ Dipartimento di Elettronica Informazione e Bioingegneria, Politecnico di Milano Milano Italy; ^3^ Department of Medical Physics and Department of Radiotherapy and Oncology Ludwig Maximilian University of Munich Munich Germany; ^4^ International Institute for Accelerator Applications, University of Huddersfield Huddersfield UK; ^5^ Clinical Radiotherapy Unit, Centro Nazionale di Adroterapia Oncologica Pavia Italy; ^6^ Division of Radiology, Istituto Europeo di Oncologia Milano Italy; ^7^ Division of Radiotherapy, Istituto Europeo di Oncologia Milano Italy; ^8^ Facoltà di Medicina e Chirurgia Università degli Studi di Milano Milano Italy; ^9^ Medical Physics Unit, Centro Nazionale di Adroterapia Oncologica Pavia Italy

**Keywords:** pancreatic cancer, PT immobilization, 4D MRI, pancreas segmentation, pancreas motion

## Abstract

Particle therapy (PT) has shown positive therapeutic results in local control of locally advanced pancreatic lesions. PT effectiveness is highly influenced by target localization accuracy both in space, since the pancreas is located in proximity to radiosensitive vital organs, and in time as it is subject to substantial breathing‐related motion. The purpose of this preliminary study was to quantify pancreas range of motion under typical PT treatment conditions. Three common immobilization devices (vacuum cushion, thermoplastic mask, and compressor belt) were evaluated on five male patients in prone and supine positions. Retrospective four‐dimensional magnetic resonance imaging data were reconstructed for each condition and the pancreas was manually segmented on each of six breathing phases. A k‐means algorithm was then applied on the manually segmented map in order to obtain clusters representative of the three pancreas segments: head, body, and tail. Centers of mass (COM) for the pancreas and its segments were computed, as well as their displacements with respect to a reference breathing phase (beginning exhalation). The median three‐dimensional COM displacements were in the range of 3 mm. Latero–lateral and superior–inferior directions had a higher range of motion than the anterior–posterior direction. Motion analysis of the pancreas segments showed slightly lower COM displacements for the head cluster compared to the tail cluster, especially in prone position. Statistically significant differences were found within patients among the investigated setups. Hence a patient‐specific approach, rather than a general strategy, is suggested to define the optimal treatment setup in the frame of a millimeter positioning accuracy.

PACS number(s): 87.55.‐x, 87.57.nm, 87.61

## I. INTRODUCTION

Pancreatic cancer has a high incidence and mortality rate worldwide. It is considered to be amongst the 10 most common tumors affecting the population of the United States, with the number of expected deaths in 2015 equaling approximately 80% of the estimated new cases.^(1)^ One of the main reasons for such a high death rate and unfavorable prognosis is related to late diagnosis; the first symptoms appear after approximately a decade of pancreatic carcinogenesis, when the tumor has reached an advanced stage.^2^, ^3^ As a consequence, only 20% of pancreatic cancer patients are candidates for surgery,^(4)^ as the majority of pancreatic cancer patients show locally advanced or metastatic disease. Locally advanced pancreatic lesions involve the main pancreatic vessels and retroperitoneum that can lead to incomplete resection,^(5)^ with associated poor survival. Novel radiation therapy techniques with accelerated ion beams have shown positive therapeutic results in local control of locally advanced pancreatic cancer.^6^, ^7^, ^8^, ^9^

The aim of conformal radiation therapy is the selective dose delivery to the target, with the maximal sparing of surrounding organs at risk (OARs). The use of ion beams in particle therapy (PT) enables dose distributions with higher physical and radiobiological selectivity compared to conventional photons. Such an increased dose conformity to the target may yield benefits in terms of enhanced efficacy and reduced side effects of the treatment.^7^, ^10^, ^11^, ^12^, ^13^, ^14^ However, this geometrical advantage is associated with a strong sensitivity to inconsistencies between the treatment plan and the actual treatment scenario. As a matter of fact, the abdominal region is affected by both inter‐ and intrafraction anatomical changes, which need to be considered in order to grant the optimal dose distribution.^(15)^ The impact of these anatomical variations is strictly related to size, shape, and anatomical site of the lesion. Targets placed in the pancreas are particularly sensible to such inconsistencies, due to the morphology of the pancreas itself, the proximity of OARs (duodenum, large and small bowel, stomach, kidneys, and liver^5^, ^16^, ^17^), and the various sources of abdominal motion. The pancreas is a glandular organ characterized by reduced thickness (2–3 cm) and height (4–5 cm), relative to its overall length (17–20 cm) and a multisegmental shape, that is typically subdivided into head, body, and tail.^(18)^ This makes pancreas localization sensitive to displacements perpendicular to the axes of the segments, and complicated by possible differences in motion between segments. Moreover, the pancreas and OARs motions vary according to prone or supine patient position.^(19)^ All these features make the pancreas a particularly challenging treatment site, thus requiring appropriate techniques for describing, handling, and mitigating pancreas motion.

In particle therapy, specific devices are dedicated to patient movement limitation during treatment delivery. Such devices should be optimized in order to handle both inter‐ and intrafraction organ motion. Interfraction variations are determined by anatomical or physiological modifications, which potentially could be managed with pretreatment in‐room imaging and replanning tools.^(20)^ Conversely, intrafraction variations are mainly introduced by periodic anatomical changes, such as breathing motion, especially in the superior–inferior (SI) direction.^21^, ^22^, ^23^, ^24^ Specific treatment planning schemes have been designed in order to account for breathing motion.^(25)^ Respiratory gated particle irradiation, in particular, entails the delivery of the planned dose in correspondence of a predefined respiratory gate featuring the highest motion stability and repeatability of the anatomopathological configuration. It has been demonstrated that this technique allows dose escalation in the target and increases the effectiveness in local control of locally advanced pancreas cancer.^26^, ^27^ Furthermore, respiratory correlated imaging techniques greatly support respiratory motion management.^(25)^ Such imaging approach is referred to as four‐dimensional or 4D imaging, and it can be applied to computerized tomography (CT) as well as to magnetic resonance (MR) imaging. Four‐dimensional CT and MR imaging yield a time‐resolved volumetric dataset composed by images describing the different breathing phases along the complete respiratory cycle. Pancreas motion has been investigated with the purpose of radiation therapy (RT) treatment with both CT and MR imaging.^21^, ^22^, ^23^, ^24^, ^28^ However, those studies mainly focused on the range of motion specific to the lesion rather than characterizing pancreas motion in presence of typical RT/PT setup immobilization devices.

The purpose of this work was to quantitatively assess the effects of patient position (prone, supine) combined with different immobilization devices (vacuum cushion, thermoplastic mask, and compressor belt) on pancreas motion. Four‐dimensional MR images were used to quantify the respiratory correlated movements of the whole gland, as well as of its anatomical segments (head, body, and tail). The ultimate goal of this study was to determine whether general guidelines can be adopted for an optimal patient setup in pancreas particle therapy, or if a patient‐specific assessment of respiratory motion mitigation strategies is to be preferred.

## II. MATERIALS AND METHODS

### A. Patient population

Four‐dimensional MR analysis of pancreas motion was performed on each of five male patients (20, 40, 51, 50, and 66 years old). The MR data were acquired between March 2014 and November 2014 in the framework of an ethics committee‐approved research protocol, and the patients provided written informed consent. The candidates were selected among patients without pancreas cancer, undergoing particle therapy treatment in the abdomen–pelvis region at our Institution, without evidence of contraindications for MR exam, compliant and capable of undergoing all the study procedures.

The present work was designed as a preliminary study, and patient population size and heterogeneity (with respect to the patient age) aimed to represent a sufficient sample to investigate whether general positioning guidelines can be applied in pancreas PT treatment setup definition.

### B. Clinical study protocol

**Figure 1:**
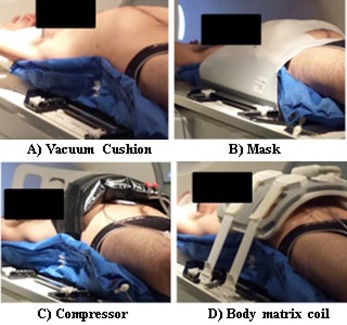
Example of the MR investigated patient setups for supine positioning. The patient was prepared on the MR couch with the specific treatment positioning device (respectively, (A), (B), (C)). The body matrix coil was finally placed on the patient before the beginning of the MR exam (D).

Individualized vacuum cushions are commonly applied as part of standard particle therapy setup strategies in order to increase the interfraction reproducibility of patient positioning. It is usually combined with other immobilization systems, such as a thermoplastic mask and/or an abdominal compressor belt, for motion mitigation when subdiaphragmatic anatomical structures are involved in the irradiation. The different combinations of immobilization strategies investigated in this study (see Fig. 1) were: (a) vacuum cushion, (b) vacuum cushion+thermoplastic mask, and (c) vacuum cushion+compressor belt.

Several studies have demonstrated significant effects of the above‐listed immobilization devices in intrafraction motion mitigation,^21^, ^23^, ^29^, ^30^ the unconstrained condition was therefore not included in the study, since our goal was to quantify the level of immobilization in the abdomen–pelvis region as a function of patient setup position and selected immobilization devices. It should be noted that the condition “vacuum cushion” cannot be considered an unconstrained condition, since the MR scan was performed with the body matrix coil slightly tightened to the patient thorax and back (supine and prone position, respectively), mimicking the effects of a mild restraining belt.

Prior to MR acquisitions, each patient was requested to drink 200 ml of water for enhancement of contrast between the duodenum and pancreas head, thus facilitating pancreas segmentation.^(23)^ The sequence of the investigated constrained conditions was the following: vacuum cushion, thermoplastic mask, and compressor belt. The vacuum cushion and thermoplastic mask were modeled on the patient in order to immobilize the abdominal region. At the beginning of the imaging procedure, patient‐specific devices were prepared on MR couch and the patient was properly positioned. The compressor belt was tightened, relying on patient's perception. For each patient, MR scans were acquired for all the three immobilization devices (Fig. 1) in supine and prone positions. Image data for the supine and prone positions were acquired on different days, in order to avoid prolonged scanning sessions. The duration of each (prone/supine) MR scanning session was approximately 45 min.

### C. MR image acquisition and 4D reconstruction

MRI scanning was performed with a 3 Tesla scanner (Magnetom Verio, Siemens Healthcare, Erlangen, Germany) using the six‐element body matrix coil (Fig. 1(D)), and patient‐specific adjacent elements of the spine matrix coil.

The same MR protocol was performed for each combination of immobilization device and patient position, separated by breaks for changing the patient immobilization system. In the MR protocol, the volume of interest was localized by means of a conventional coronal MR localizer sequence, and a T2‐weigthed axial spin‐echo acquired with breath‐hold triggering (20 slices with 4 mm slice thickness).

Dynamic MR images of the pancreas were acquired with a TrueFISP (True Fast Imaging with Steady‐state Precession) sequence with the following acquisition parameters: TE/TR/Tslice 1.63/3/216.97 ms, Flip Angle 40°, Grappa factor 2, bandwidth 898 Hz per pixel, field of view (FoV) $450\times 337\times 100\;{\text{mm}}^{3}$, acquisition voxel size $3\times 2.3\times 4\;{\text{mm}}^{3}$ interpolated by zero padding to a reconstructed voxel size of $1.17\times 1.17\times 4\;{\text{mm}}^{3}$, reconstructed image matrix 384×288×25, and interleaved slice acquisition order. Thirty measurements of each of the 25 axial slices were acquired during patient free‐breathing, corresponding to an overall scan time of 162 s.

A 4D MRI dataset consisting of image volumes at 6 respiratory phases was reconstructed by means of image‐based retrospective sorting of the acquired dynamic MR images. According to Paganelli et al.^(31)^ a reference volume was constructed by selecting the distinct slices with closest mutual information. An internal anatomical surrogate of the respiratory signal was then obtained by computing the mutual information between the individual images of the dynamic acquisition and the reference volume. Images falling within the 6 respiratory phases were then identified and grouped to form the complete 4D MR volume reconstruction. This method has been previously used to produce 4D MRI of the liver for both sagittal and axial MR acquisitions.^(32)^

### D. Pancreas segmentation

The pancreas was manually segmented from the reconstructed 4D MR images for each of the immobilization and patient position configurations by a trained operator, and the results revised by an expert radiologist. The contouring procedure was performed on a three‐dimensional (3D) image processing software platform (AMIRA, FEI Visualization Sciences Group, Hillsboro, OR).

**Figure 2:**
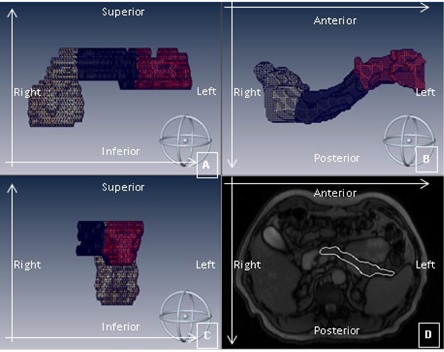
Coronal (A), axial (B), and sagittal (C) views of pancreas manual segmentation. Manual segmentation map is partitioned in the three pancreas segments based on the k‐means clustering algorithm: head (yellow cluster), body (black cluster), and tail (red cluster). Illustration of a manual contouring (D) of the pancreas in an axial slice of the 4D MR volume. Coordinate axes are reported on each panel.

An algorithm implemented in MATLAB (Version: R2010a, MathWorks, Inc., Natick, MA) was used to divide the segmented binary pancreas map into three subvolumes representative of the head, body, and tail segments of the pancreas. The subdivision method was based on the k‐means clustering algorithm (Fig. 2)^(33)^ that was applied to the 3D spatial coordinates of the pancreas map and required three initialization centroids as input. For the first breathing phase (phase 1, end inhalation), the centroids were obtained by a random initialization. The cluster centroids obtained for a given breathing phase were then adopted as initialization centroids for the subsequent phase. Finally, the algorithm was repeated on the first breathing phase, using the centroids from the last phase, in order to correct for the random initialization effects. The resulting subvolumes, as well as the whole pancreas segmentation, were used for motion analysis. In the following they will be referred to as head, body, and tail pancreas segments and whole pancreas.

### E. Segmentation reliability index

The contouring of the pancreas and the definition of its segments within different breathing phases in the 4D MR dataset represented a critical issue for this work, with potentially high impact in the motion analysis. Therefore, two quantitative indexes were defined in order to evaluate the reliability of both manual contouring and pancreas subvolume identification. In particular, it was assumed that robust segmentations would highlight approximately the same shape and volume within the different breathing phases as the pancreas motion was considered a nearly rigid displacement.

The first index was based on Pearson's correlation between integral map profiles for different breathing phases. This index allowed robust evaluations among all the three image coordinates while preserving the information represented in the whole segmentation map. The integral map profiles were calculated by summing voxel intensities along each image coordinate axes: latero–lateral (LL), anterior–posterior (AP), and superior–inferior (SI).

(1) $${\text{profile}}_{i}=\sum_{j=1}^{J}\sum_{k=1}^{K}\text{segmentation}$$

where indexes *i, j, k* iterate along each coordinate axes in order to construct the integral *profile* of the specific i‐th image coordinate, for the considered *segmentation* map (i.e., pancreas, head, body, and tail maps).

The integral map profiles were rigidly registered onto a reference breathing phase (as defined below) according to their approximate center‐of‐mass (COM) displacement in order to compensate the effects of the rigid amount of motion due to breathing. The Pearson's correlations between the profiles of each breathing phase and the other breathing phases were computed. Such index reflects the shape accuracy in manual and automatic segmentations, since the embedded rigid motion effect was minimized through the profile registration process. The median values and interquartile ranges of the Pearson's correlation values related to each breathing phase were then computed, obtaining an index for each of the 6 breathing phases. The same evaluation was also performed for head, body, and tail pancreas segments.

The second reliability index was based on the consistency between segmented volumes on different breathing phases. The volume consistency was computed as the median values and interquartile ranges of the volume variations among breathing phases, calculated as the percentage difference with respect to the maximum volume. Volume consistency was evaluated also for pancreas segments.

### F. Quantification of pancreas motion

Pancreas motion analysis was performed on the 4D MR dataset of each immobilization/patient position combination except for the supine compressor positioning configuration in Patient 3, because of inadequate image quality. In particular, the image contrast among tissues was not sufficient to enable a reliable pancreas segmentation in the caudal portion of the acquired volume. This image effect could be likely attributed to a signal worsening derived from a malfunctioning of the MR scanner system, or the cranial sliding of the body matrix coil over the compressor belt surface toward the patient chest.

In order to determine a reliable reference breathing phase to allow robust MR comparison among different patient positions and immobilization combinations, we examined the stability of the center of mass estimates. The three‐dimensional (3D) COM was computed for each breathing phase, both for the whole pancreas and pancreas segments. The COM stability within the breathing cycle was calculated for each phase as follows:

(2) $$COM\;\text{stability}=\frac{\left(|COM_{ph–1}–COM_{ph}|+|COM_{ph+1}–COM_{ph}|\right)}{2}$$

The COM stability index reflects the absolute mean COM displacement relative to the adjacent breathing phases. The breathing phase with maximal stability (minimum of the COM stability index) was selected as the reference phase for motion quantification, as described in the following paragraph.

The 4D pancreas motion was expressed as COM displacement relative to the reference breathing phase. Note that in the context of gated particle therapy treatments, this quantification evaluated the pancreas motion with respect to the selected treatment planning position (being the gate with the highest organ position stability).

The hypothesis about a preferable positioning setup was tested on the five patients. Statistically significant differences in COM motion patterns of the whole pancreas and its segments were evaluated by means of the nonparametric Friedman's test. In particular, a nonparametric equivalent of Fisher's least significant difference method (as proposed by Conover^(34)^) was applied as a post hoc analysis^(35)^ in order to assess the presence of statistically relevant differences among the investigated combinations of patient positioning (prone/supine), immobilization devices, and pancreas segments.

## III. RESULTS

### A. Segmentation reliability index

Table 1 summarizes, for all patients, the median values and interquartile ranges of the segmentation reliability indexes for the pancreas and related anatomical segments.

Pearson's correlation applied to the evaluated integral map profiles was above 0.7 for each patient and each breathing phase. Pearson's correlation values among SI profiles were slightly lower with respect to the ones found for LL and AP profiles. In particular, phase 1 and phase 6 (corresponding to inhalation) showed lower median SI profile correlation values with respect to phase 3 (corresponding to exhalation). The computed inhalation phases percentage differences with respect to phase 3 were computed and the following results were obtained (phase 1 and phase 6): 1.88% and 1.71% for pancreas segmentation, 1.05% and 1.32% for head segmentation, 1.33% and 1.90% for body segmentation, 1.70% and 1.09% for tail segmentation, respectively.

Median values for volume consistency showed high reliability for both manual segmentation and partitioning of the pancreas, even though body and tail segments reported higher median values and interquartile ranges (i.e., lower volume consistency) compared to the whole segmentation and head segment.

**Table 1:** Pancreas and clusters segmentation reliability indexes. Pearson's correlation median and interquartile values and volume and volume consistency grouped by the evaluated segmentations: pancreas, and head, body, tail clusters.

	*Pearson's Correlation LL Profile*	*Pearson's Correlation AP Profile*	*Pearson's Correlation SI Profile*	*Volume (cc)*	*Volume Consistency (%)*
Pancreas	0.991±0.018	0.997±0.005	0.952±0.077	61.334±20.407	5.912±4.790
Head	0.997±0.005	0.997±0.004	0.976±0.047	26.410±12.758	7.762±4.761
Body	0.992±0.015	0.994±0.009	0.968±0.052	17.134±5.040	9.431±5.806
Tail	0.992±0.015	0.995±0.007	0.973±0.051	16.288±7.353	12.280±10.165

### B. Phase stability

**Figure 3:**
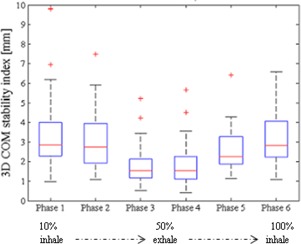
3D COM stability index, with box plots of 3D pancreas COM stability computed for each breathing phase, considering all patients data. On each box, the central mark is the median value, the edges of the box are the 25th and 75th percentiles, the whiskers extend to the most extreme data points not considered outliers. Data points are drawn as outliers if they are larger than $q_{3}+1.5\times (q_{3}-q_{1})$ or smaller than $q_{1}-1.5\times (q_{3}-q_{1})$, where $q_{1}$ and $q_{3}$ are the 25th and 75th percentiles, respectively.

Figure 3 illustrates the 3D COM stability associated with each breathing phase. Phase 3 and phase 4 are related to exhalation, while phase 1 and phase 6 to inhalation. Thus the COM stability index reflects the breathing cycle trend with lower index values for exhalation relative to inhalation. Indeed, phase 3 showed the highest 3D stability in each motion direction, supporting its adoption as the reference breathing phase for pancreas motion analysis. We also note that the selected reference breathing phase exhibited higher COM stability indexes for SI and LL directions than for the AP direction.

The overall COM stability values (median value±interquartile range) across breathing phases for prone and supine positions, respectively, were as follows: 1.83±1.93 mm and 1.65±1.27 mm (LL direction), 0.42±0.30 mm and 0.53±0.34 mm (AP direction), and 1.09±0.75 mm and 1.28±1.02 mm (SI direction).

When classified by immobilization strategy, the overall COM stability values across breathing phases for vacuum cushion, thermoplastic mask, and compressor, respectively were as follows: 1.06±1.24 mm,0.71±0.89 mm, and 0.70±1.13 mm (LL direction), 0.51±0.75 mm,0.44±0.28 mm, and 0.47±0.50 mm (AP direction), 1.04±0.94 mm,0.77±0.60 mm, and 0.97±0.66 mm (SI direction).

The Friedman test did not show any statistically significant differences in COM stability under any of the investigated conditions.

### C. Pancreas motion

**Figure 4:**
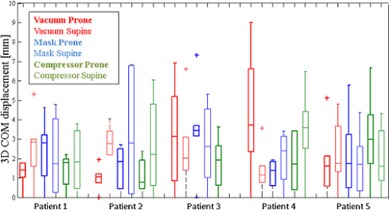
Pancreas 3D COM motion, with box plots of 3D pancreas motion computed for each patient and each positioning setup (i.e., vacuum cushion, mask, compressor in prone and supine configuration). Each box shows median value, 25th and 75th percentiles, as described for Fig. 3.

Pancreas motion was analyzed for each patient in terms of COM displacement with respect to the reference breathing phase (phase 3). Figure 4 depicts 3D pancreas COM displacements for each patient under all the investigated configurations, in each breathing phase.

**Figure 5:**
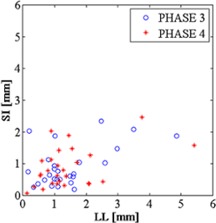
Scatter plot representing phase 3 and phase 4 COM stability, comparing LL and SI motion directions of the whole pancreas map.

Pancreas COM displacements for each motion direction are summarized in Table 2. Globally the median values for each motion direction were below 2.5 mm, with higher values found for the LL or SI directions with respect to AP (Fig. 5). Each patient showed an individual pancreas motion pattern, which was not globally consistent as a function of prone/supine position or immobilization systems.

**Table 2:** Pancreas COM motion for the investigated patient setup, considering each motion direction. COM motion data are reported for each patient and each investigated positioning (vacuum cushion, mask, compressor in prone and supine configurations). Median values and interquartile ranges were recovered from breathing phase COM motion. Each COM motion is intended with respect to breathing phase 3.

		*PRONE*			*SUPINE*		
		*LL (mm)*	*AP (mm)*	*SI (mm)*	*LL (mm)*	*AP (mm)*	*SI (mm)*
Patient 1	Vacuum	0.82±1.69	0.08±0.58	0.20±1.67	−0.55±1.84	0.80±1.07	0.34±2.28
	Mask	−2.36±2.37	−0.37±0.61	−0.67±1.75	1.55±2.94	0.07±0.52	0.37±2.15
	Compressor	0.21±2.12	0.09±0.49	0.28±1.34	−0.02±1.01	0.31±0.59	1.29±3.21
Patient 2	Vacuum	−0.47±1.05	−0.07±0.66	−0.42±1.22	0.28±0.75	0.10±0.51	0.56±1.00
	Mask	−1.38±1.53	−0.30±0.68	−0.68±0.85	−0.26±1.99	0.50±0.68	0.07±1.40
	Compressor	−0.08±0.87	−0.06±0.32	−0.04±0.60	−1.66±4.30	0.57±1.06	0.45±0.66
Patient 3	Vacuum	0.20±3.07	−0.11±0.77	2.38±3.62	0.19±2.26	0.36±0.41	1.81±1.30
	Mask	1.69±3.05	0.50±0.43	1.88±1.71	−0.26±1.26	0.25±1.31	1.33±1.39
	Compressor	0.34±2.62	0.21±0.61	0.55±0.93	‐	‐	‐
Patient 1	Vacuum	1.99±2.63	−0.06±0.29	2.07±3.69	−0.32±1.48	−0.13±0.67	−0.12±0.72
	Mask	0.28±1.87	−0.09±0.49	−0.04±0.66	−0.85±3.13	−0.02±0.15	0.29±0.93
	Compressor	0.06±4.66	0.21±0.44	−0.03±0.22	−0.35±4.93	−0.18±1.09	−0.75±3.62
Patient 5	Vacuum	−0.26±2.63	0.10±0.78	−0.04±1.31	−0.06±1.95	0.59±2.11	1.03±1.48
	Mask	0.17±1.15	0.43±1.10	0.64±1.38	−1.63±2.46	−0.14±0.51	−0.06±0.51
	Compressor	−1.73±4.16	0±1.45	0.33±2.06	−0.91±1.09	0.52±1.21	0.78±2.31

**Figure 6:**
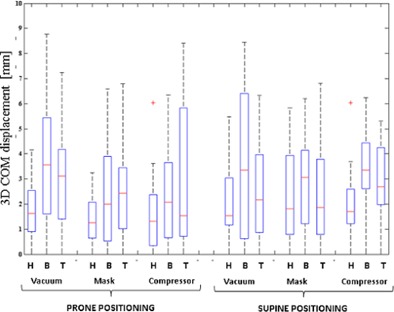
Pancreas cluster 3D COM motion, with box plots of 3D pancreas clusters motion computed for each pancreas cluster and each positioning setup (i.e., vacuum, mask, compressor in prone and supine configuration). Each box shows median value, 25th and 75th percentiles, as described for Fig. 3. Head, body, and tail pancreas clusters are, respectively, indicated as H, B, and T.

Motion of the pancreas segments for each patient is described in Fig. 6 for all the investigated positioning. Considering all the patients' data together, LL and AP motions of the individual pancreas segments were consistent with the corresponding whole pancreas motion. This result was confirmed by Friedman's tests and related post hoc analysis (Table 3). Along the SI direction however, three patients (2, 4, and 5) showed statistically significant differences between motion of the whole pancreas and its individual segments, though absolute displacements were in the range of few millimeters.

**Table 3:** Statistically significant differences resulted from Conover post hoc multiple comparisons tests associated to Friedman's test among COM motion of prone/supine positioning, immobilization systems (vacuum, mask, and compressor), and pancreas segmentations (pancreas, head, body, and tail). Any statistically significant difference is marked with a symbol (X, •, or |), hence no symbols means no statistically relevant differences found with all the tested comparison groups. Different symbols among evaluated data (column) mean a statistically significant difference was observed between related investigations (rows). Where multiple symbols are depicted, investigations with the same symbol should be considered homogeneous.

	*Patient 1*				*Patient 2*				*Patient 3*				*Patient 4*				*Patient 5*				*All Patients*			
	*LL*	*AP*	*SI*	*3D*	*LL*	*AP*	*SI*	*3D*	*LL*	*AP*	*SI*	*3D*	*LL*	*AP*	*SI*	*3D*	*LL*	*AP*	*SI*	*3D*	*LL*	*AP*	*SI*	*3D*
Prone/Supine	X	X	X			X	X	X					X				X		X			X	X	X
Vacuum		X	X				X			X	X		X		X							X		X
Mask	X	|	|				|			|			|		|	X						|		|
Compressor	|	X	X				X			X	|		|		|	|			X	X			X	X
Pancreas				X	X	X	X•	X•				X|			X	X			X	X			X	X
Head				X	X	X|	|	•		X		X				X			X	X			X	•
Body				|	|	|	X|	|		|		|			X	X			X	|			X	|
Tail				X	X	•	•	X		|		|			|	•			|	X			|	|

### D. Statistical analysis

Statistically significant differences were investigated by the Friedman test and related post hoc analysis among evaluated patient positioning setups and pancreas clusters COM motion, as summarized in Table 3. In particular, the hypothesis of statistically relevant differences was tested separately for the following COM motion comparisons: prone vs. supine (1st row); vacuum cushion (2nd row) vs. thermoplastic mask (3rd row) vs. compressor belt (4th row); pancreas (5th row) vs. head (6th row) vs. body (7th row) vs. tail (8th row) segmentations.

The three comparisons were tested for each patient and each motion direction, thus each column of Table 3 reports the results of the specific comparison. Specifically:

i) Each statistically relevant difference was marked with a symbol along the column (e.g., Patient 2 SI: prone significantly different from supine);

ii) Rows marked with the same symbol belong to an homogeneous group along the column (e.g., Patient 2 SI: vacuum significantly different from mask and homogeneous with compressor);

iii) Rows marked with multiple symbols are homogeneous with the rows marked with at least one of its own symbols (e.g., Patient 2 SI: pancreas homogeneous with body and tail and different from head);

iv) No symbol means an homogeneous behavior (e.g., Patient 1 SI: pancreas homogeneous with the segments).

Data from Patient 3 in supine position with compressor belt is missing, therefore Patient 3 prone/supine statistical tests were based on vacuum cushion and mask data. Similarly for Patient 3, vacuum/mask/compressor multiple comparisons were based on prone data, and all patients' analysis was based on all prone patients data and supine data from Patients 1, 2, 4, and 5.

Considering all the patients' data together, statistically significant differences were observed in at least one motion direction for all the considered tests (prone/supine position, immobilization systems, and pancreas maps multiple comparisons), except for LL motion direction which did not show any statistically significant differences (no symbol marking the related LL column in Table 3).

Considering the complexity of the results, it is worth evaluating each patient separately. The statistical tests showed different results both inter‐ and intrapatient. Within the same patient, different statistically relevant differences were reported for the three motion directions and 3D motion, hence outlining different motion pattern in the context of the specific comparison. In particular, the effectiveness in the motion reduction varies in the three coordinates with respect to the evaluated comparisons (prone/supine and immobilization systems). Similarly, the Friedman test and related post hoc analysis showed complex results for pancreas segments' motion, as represented in Table 3, where multiple symbols could even mark the same investigation (being investigations with the same symbol considered homogeneous). The pancreas segments showed different motion patterns, thus resulting in different outcomes for the motion directions.

## IV. DISCUSSION

Particle therapy of pancreatic lesions requires confronting several important clinical issues relating to target definition and efficient treatment delivery. Targeting the pancreas is considered a critical ballistic challenge because of its proximity to radiosensitive vital organs including the duodenum and stomach. Highly accurate pancreatic lesion localization should be sought in order to maximize PT outcome and minimize gastrointestinal toxicity. Since the pancreas undergoes significant organ motion, mainly induced by breathing, pancreas motion characterization within the actual treatment setup is also a fundamental requirement for patient positioning optimization. Different patient immobilization systems are commercially available for patient positioning in PT. The quantification of pancreas residual motion as a function of the efficacy of these immobilization systems is believed to help clinicians in the definition of patient setup and treatment planning margins.

Our study aimed to characterize the motion of the pancreas and its segments by means of 4D retrospective MRI. Four‐dimensional MRI provides volumetric information, which could more completely characterize organ motion compared to other MR techniques, such as cine‐MRI.^(36)^

According to our evaluations, manual pancreas segmentation was highly consistent across respiratory phases. Median values and interquartile ranges for pancreas segmentation volume were consistent with previous investigation.^(37)^ Similarly, the partitioning of the whole pancreas segmentation into its anatomical segments by means of the k‐means algorithm turned out to be consistent within the breathing cycle. We expect that such partitioning can be useful in clinical practice, as the obtained subvolumes represent the different pancreatic lesion site. Therefore, important information related to the pancreatic cancer motion pattern may be derived from the characterization of pancreas segments' motion.

The COM phase stability was evaluated in order to define a reference breathing phase for pancreas motion comparison among different MR exams. This quantification led to interesting considerations in the context of gating particle treatments. These treatments, performed in presence of clinically relevant organ motion, aim to deliver the planned dose in a predefined respiratory gate. Phase stability is thus an important parameter for the selection of an appropriate gating window. The beginning of exhalation (phase 3) displayed the highest phase stability. This result is partially consistent with previous studies assessing the end exhale as the most stable phase.^22^, ^23^ The COM stability index in the LL direction was comparable, or even higher, with respect to the one obtained for the SI direction. Both LL and SI should be considered as critical pancreas motion directions; hence, both their phase stability range of motion should be evaluated for treatment planning purposes.

Pancreas range of motion was computed with respect to the exhale phase, which demonstrated the highest stability within the presented study. This breathing phase would most likely correspond to the treatment gate in gated PT treatments. The motion characterization thus provided information regarding pancreas displacements with respect to the pancreas treatment planning position. While using three common immobilization devices for patients in prone and supine positions, we found pancreas ranges of motion that were globally lower than what reported in previous studies, where no immobilization systems were applied, and maximal motion values on the order of 20 mm were found in the SI direction.^21^, ^23^, ^29^, ^30^ In keeping with our results, a prior study investigating the effectiveness of immobilization systems reported superior–inferior motion of 4.4 mm for abdominal organs with the compressor device, thus proving a significant motion reduction.^(38)^ In the current study we found median motion in SI direction below 2.5 mm, and similar motion in the LL direction. LL and SI range of motion and COM stability index results were both higher than in AP direction, characterized by median values below 1 mm. The effectiveness of immobilization systems for motion reduction seemed lower in LL direction than in SI and AP directions.

We also observed slightly lower displacements for pancreas head cluster relative to tail cluster, especially in prone position. This is in agreement with previous studies, which reported lower motion values for the pancreas head segment with respect to the tail segment.^19^, ^39^

This study focused on the analysis of pancreas motion in presence of different immobilization systems. The baseline condition of unrestrained motion was not characterized since prior studies have documented the substantial reduction in abdominal organ displacement provided by immobilization. Therefore, we did not evaluate the overall motion reduction capability of the immobilization devices, but focused on the relative differences in displacement control among the different immobilization strategies. Our results show that the use of vacuum cushion resulted in pancreas motion comparable to the other immobilization devices. As specified in the methods section, this outcome could be mainly explained by the presence of the body matrix coil during MR scanning, resulting in a mild compression on the patient thorax. Unfortunately, the applied degree of motion limitation could not be quantified within the presented study, since further CT scans (without body matrix coil compression) would be necessary for accurate pancreas COM motion assessments for vacuum cushion setup. In the framework of our study, the CT extra scan was unfeasible, especially considering the delivery of ionizing radiation to the patient. A CT scan without treatment setup immobilization systems would not be justifiable by any clinical advantages to the patient and it was not included in the presented study. Although, it is worth noting that the body matrix coil was applied for all the MR exams, thus its effect in motion limitation can be considered homogeneous among the different investigations, and the vacuum cushion can be reasonably considered as the major responsible for motion restriction, allowing fair comparisons with the other immobilization strategies.

Statistically significant differences were found among the investigated patient positions within the same patient. All patients (except for Patient 3) showed statistically significant differences between the prone and supine positions for at least one motion direction. However, no general recommendation on patient position can be made from the present study as two patients (Patient 1 and Patient 2) had a lower range of motion in prone position, consistently with previous study,^(19)^ while for the other two patients (Patient 4 and Patient 5) the opposite was found. Similarly, although statistical analysis allowed us to highlight possible optimal solutions for individual patient setup and immobilization, our analysis suggests that the observed interpatient variability, with 3D pancreas motion up to 3 mm as a function of patient position and applied immobilization device, does not support the hypothesis of a unique optimal generic patient setup. Furthermore the statistical test results outlined different behavior in motion reduction with respect to the motion direction, for each evaluated positioning strategy.

With regard to the study results, and the PT requirements, a patient‐specific setup investigation is justified since it could lead to a relevant enhancement of the treatment quality. Nonetheless, such time‐consuming procedure could hardly be introduced in the routine clinical practice. Therefore, at first, the setup could be defined on the basis of established clinical and dosimetric parameters, and subsequently, perform a preliminary fast MR acquisition oriented to a careful evaluation of the obtained pancreas range of motion, following the procedure described in the Materials and Methods section. In case of suboptimal patient immobilization, further setup investigations would be highly recommended before beginning the proper treatment simulation and planning process. A relevant advantage could be gained with a different positioning.

On the other hand, the increase of the sample size could lead to important indications towards pancreatic patients' positioning, and the patient‐specific conclusions that emerged from this preliminary study could be the basis for the design of future investigations. In particular, because of the heterogeneity of our study sample with respect to the obtained results, we would hypothesize an optimal configuration depending on patient characteristics (both clinical and dosimetric).

Finally it is worth noting that pancreas motion assessment should be integrated with further motion related observation in order to optimize PT patient setup. Specifically, motion characterization of surrounding OARs should be also taken into account, since dose distribution is strongly affected by tissues along the beam path. OARs and target dose volume histograms (DVHs) should be computed in order to assess the presence of statistically significant dose differences among evaluated positioning strategies. In the frame of a 4D MRI‐based dosimetric studies, the MR volume resolution should be defined in order to cover the entire treatment planning CT FOV, thus enabling the computation of the breathing induced motion model for the 4D planning CT reconstruction. Consequently, the DVH would be computed directly on the simulated 4D CT.

Furthermore, immobilization systems should be evaluated both for their effectiveness in motion reduction and their patient positioning reproducibility. Previous studies reported better positioning reproducibility in the pelvis and abdominal regions with vacuum + thermoplastic mask configuration compared to vacuum alone,^(40)^ but abdominal compressor reproducibility was not evaluated. Dedicated studies should be carried out in order to assess immobilization devices effectiveness in pancreas positioning reproducibility. Due to absence of ionizing radiation and better image contrast for soft tissues, MR is the recommended imaging modality for such investigations.

Similarly, the breathing cycle reproducibility represents a critical parameter to be evaluated with respect to the selected positioning strategy. Particularly, in the frame of gating treatments, it could lead to potentially inaccurate treatment gate identification, thus affecting the overall quality of the therapy. Future studies should be oriented to such investigation, and efforts towards the definition of reliable pancreas motion surrogates for accurate treatment gate identification should be attempted. Actually dedicated dose delivery strategies, such as rescanning, are devoted to mitigate this effect.

## V. CONCLUSIONS

Our study aimed at pancreas intrafraction range of motion quantification by means of 4D MRI, when typical PT immobilization systems are applied. Median pancreas COM 3D displacements were observed to be lower than 3 mm among all six evaluated patient positioning setups. Pancreas motion along LL and SI directions was higher than along the AP direction, with motions in the range of few millimeters and below one millimeter, respectively. Nonetheless particle therapy may require higher positioning accuracy for pancreas treatments. In this context, patient‐specific setup investigation is recommended, since each of the five investigated patients showed different results in terms of optimal immobilization device. Statistically significant differences within the same patient were effectively reported among the investigated setups. Similarly, the pancreatic lesion site should be considered, since the evaluation of COM displacement within pancreas clusters highlighted lower displacements for pancreas head when compared with body and tail clusters, with median 3D difference around 2 mm. Due to highly patient‐specific results, no general indications with regard to the optimal setup and immobilization strategy emerged from this study. Nonetheless, the patient‐specific conclusions derived from the presented preliminary study could be the basis for the design of future investigations, which would consider a larger and properly heterogeneous population.

Beside pancreas motion, other parameters, such as setup reproducibility and breathing induced OARs motion, with associated dosimetric impacts, should be considered for patient positioning optimization.

## ACKNOWLEDGMENTS

Authors would like to thank CNAO radiologic technologist staff for the technical support in MR data acquisition.

## COPYRIGHT

This work is licensed under a Creative Commons Attribution 3.0 Unported License.
